# Effect of fermented spent instant coffee grounds on milk productivity and blood profiles of lactating dairy cows

**DOI:** 10.5713/ajas.18.0846

**Published:** 2019-02-14

**Authors:** Yongjun Choi, Jongsu Rim, Honggu Lee, Hyunchul Kwon, Youngjun Na, Sangrak Lee

**Affiliations:** 1 Department of Animal Science and Technology, Konkuk University, Seoul 05029, Korea

**Keywords:** Spent Instant Coffee Ground, Fermentation, Milk Production, Milk Composition, Lactating Dairy Cow

## Abstract

**Objective:**

This study was conducted to evaluate the fermentation characteristics under low mesophilic temperature of spent instant coffee ground (SICG) and to estimate the effect of fermented SICG (FSICG) as alternative feed ingredient on milk productivity of dairy cows.

**Methods:**

In the fermentation trial, fermentation of SICG was performed to investigate changes in characteristics using the microbial mixture (*Lactobacillus plantarum*, *Saccharomyces cerevisiae*, and *Bacillus subtilis* = 1:1:1) for 21 days at 20°C under anaerobic conditions. Molasses was added at 5% of dry mass. In the animal trial, eighteen Holstein Friesian cows were used to evaluate the nutritive value of the FSICG which was fermented for 14 days under the same condition as the fermentation trial.

**Results:**

In the fermentation trial, the dry matter (DM) and organic matter content linearly decreased with fermentation time (p<0.001 and p = 0.008, respectively). The acid detergent insoluble nitrogen content linearly decreased with fermentation time (p = 0.037). The microorganism counts linearly increased for *Lactobacillus plantarum*, *Saccharomyces cerevisiae*, and *Bacillus subtilis* across fermentation time (p<0.001). In the animal trial, the DM intake of the control and FSICG treatment were not significantly different, as were milk yield, 4% fat corrected milk, fat-protein corrected milk, and feed to milk conversion content. Fat, protein, lactose, non-fat solids, milk urea nitrogen, and somatic cell counts were also not significantly different in milk composition between treatments.

**Conclusion:**

FSICG should be considered a sufficient substitute for cottonseed as a feed component, and 5% DM of a dietary FSICG level was appropriate for dairy cow diets.

## INTRODUCTION

Coffee is the second most traded item in the world next to oil [1]. The increase in coffee consumption has caused an increase in waste, with approximately 10 thousand tons of spent coffee grounds (SCG) produced every year in South Korea. For this reason, the recycling of SCG is important and various studies have been conducted to better utilize the SCG such as through sugar extraction, composting, and sorbent for metal ions [2]. Furthermore, previous studies have reported that coffee grounds exhibited a useful chemical composition as a ruminant feed component [3,4]. However, there are some disadvantages that may limit its use as a feed component, such as low palatability and low nitrogen digestibility [5]. Therefore, enhancing the palatability and nitrogen digestibility of SCG is needed to utilize it as a feed component.

The SCG contain various antioxidants such as caffeine, melanoidin, and polyphenols [6]. Of these antioxidants, melanoidin is produced through high temperature and pressure and is known to have low nutrition and digestibility in animals [2]. In the previous study, it was reported that molasses wastewater including melanoidin was decolorized by *Lactobacillus plantarum* (*L. plantarum*) isolated from pickle [7]. Furthermore, fermenting spent instant coffee grounds (SICG) with *L. plantarum* showed a positive effect on protein digestibility in sheep [8]. However, few studies have been conducted on the use of SCG as an animal feed in dairy cows.

The volume of production and cost of by-products is an important factor influencing the use of SCG as a feed component. Although fermentation has a known positive effect on the function of SCG, it is a factor that increases the cost of the by-product. In a previous study, fermentation was performed under anaerobic tension and mesophilic conditions [8]. These conditions might increase the cost to produce and make it less competitive as a feed component. For this reason, a study on a cheaper fermentation method is needed in order to utilize it on the farm.

Therefore, the objective of this study was: i) to evaluate the fermentation characteristics of the SICG under low mesophilic temperatures, and; ii) to estimate the effect of fermented SICG (FSICG) as alternative feed ingredient on the milk productivity of dairy cows.

## MATERIALS AND METHODS

This experiment was performed in compliance with the guidelines of the Institutional Animal Care and Use Committee at Konkuk University (Approval number: KU16139).

### Spent instant coffee grounds and fermentation process

The SICG used in the experiment originated from the factory of Dongsuh food industry (Incheon, Korea) and was stored at −20°C until commencing the experiment. The chemical composition of SICG was determined and shown in Table 1. The SICG was sterilized using an autoclave (HB-506, HANBAEK Co., Bucheon, Korea) before lab scale fermentation.

Fermentation was performed to investigate the change in characteristic and to determine fermentation period of SICG using *L. plantarum* (ATCC 14917), *Bacillus subtilis* (*B. subtilis*; ATCC 6633), and *Saccharomyces cerevisiae* (*S. cerevisiae*; ATCC 13007) in the lab scale. The microorganisms were cultured in a liquid medium and the number of microbes calculated from the growth curve of the solid medium after 24 h fermentation time using an ELISA reader (Bio-Tek, Winooski, VT, USA). The microorganisms to be inoculated were cultured in a liquid medium for 24 h and then prepared in a ratio of 1:1:1, inoculated at 1.0×10^5^ cfu/g of dry matter (DM). Fermentation of SICG was performed using a sterile plastic bag (Whirl-Pak, Nasco, Fort Atkinson, WI, USA, 10 cm×20 cm) for 21 days at 20°C in anaerobic condition following the addition of molasses. Sampling was performed at 0, 3, 6, 9, 12, 15, and 21 days.

Based on the result of lab scale fermentation, instant coffee grounds were respectively divided into 200 kg portions (approximately 60.0% DM) and each placed into a separate plastic bag (100 cm×100 cm×140 cm). This was carried out with eight replications for utilizing as a feed ingredient in the dairy cow. The fermentation was performed by inoculating 1.0×10^5^ cfu/g DM of the mixed inoculum (*L. plantarum*, *B. subtilis*, and *S. cerevisiae* = 1:1:1) for 14 days at a moisture of 70% and temperature 20°C in the anaerobic condition following the addition of molasses. After mixing the inoculum, the mixture was compressed to remove air and was flushed with carbon dioxide gas in the plastic bags to make anaerobic conditions.

Fermentation quality was evaluated by chemical compo sition, pH, volatile fatty acids (VFA) and ammonia nitrogen content after sampling. The pH was immediately estimated after sampling, the samples were stored at −20°C after pretreatment for analysis of chemical composition, ammonia nitrogen, and VFA.

### Microorganism counts

The sample was prepared by adding 450 mL distilled water containing 25% glycerine to 50 g FSICG and the supernatant collected after homogenization. The number of microorganisms was determined using the diluted supernatant with 10^−2^, 10^−3^, 10^−4^, 10^−5^, and 10^−6^ according to the method of standard plate count [9]. The number microorganisms were compared using a log_10_ scale.

### Animals and experiment design

A total of eighteen Holstein Friesian cows (body weight: 690.0 ±63.0 kg) were used during the experiment. The average temperature and relative humidity during the experiment were 9.8°C±4.3°C and 70.6%±12.7%, respectively. The average number of calves produced by experimental animals was 2.3±1.3 year, and the number of days in milk was 194.0±13.0 days. Animals were organized according to milk yield, days in milk and parity and then allotted into six sawdust-bedded pens (three head/pen) with an individual electronic feeding gate. The experimental unit was an individual animal. The treatments were basal diet (control) and FSICG (experimental), with the diet formulated according to NRC guidelines [10] (Table 2). In the experimental diet, cotton seed and cotton seed pellets in the basal diet were replaced by FSICG (Table 3). The experiment was performed using a randomized block design for six weeks (individual electronic feeding gate adaptation period, two weeks; experimental diet adaption period, four weeks; data collection period, two weeks). Experimental feeds were fed twice a day at 0900 and 1600 h *ad*-*libitum* in form of total mixed ration. The residue of the previous feeding was weighed before the next feeding. The water and mineral block were available *ad*-*libitum*.

### Milk yield and composition

Milk yield was automatically measured using a tandem milking system (Milking Parlor Auto Tandem, GEA Group Co., Düsseldorf, Germany) twice a day at 0500 and 1600 during the entire experimental period. The sample of milk was collected in 20 mL containers using the sampling port on a milking machine each week. An anticorrosive agent (Broad spectrum microtabs II, Advanced Instrument Inc., Norwood, MA, USA) was added to prevent any changes in the milk sample before being stored at 4°C until the analysis of the milk composition. The milk composition was evaluated using near-infrared spectrophotometer (Milko-scan FT 6000, Foss electric Co., Hilleroed, Denmark).

### Chemical analysis

All samples were dried in an oven (HB-503-LF, Hanbaek Scientific Technology, Bucheon, Korea) at 60°C for 48 h and they were ground and passed through a 1 mm screen with a micro hammer mill (Nr9737840, Culatti AG, Steinenberg, Switzerland). The DM, organic matter (OM), crude protein (CP), and ether extract (EE) were analyzed according to AOAC method [11]. The neutral detergent fiber (NDF) and acid detergent fiber (ADF) were analyzed with ANKOM Fiber Analyzer (A200, Ankom Inc., USA) according to method of Van Soest et al [12]. The non-fiber carbohydrate content was calculated by subtraction of CP, NDF, EE, and ash from 100. Acid detergent insoluble nitrogen (ADIN) was measured for nitrogen using ADF residues, and the nitrogen contents were determined using a distillation unit (B-324, Buchi, Flawil, Switzerland) according to method of Licitra et al [13]. The pH was obtained using pH meter (Orion Dualster-F, Thermo Fisher Scientific, Waltham, MA, USA), and ammonia nitrogen was determined according to method of Chaney and Marbach [14]. The VFA was identified by using gas chromatography (HP 6890, Agilent Technologies, Santa Clara, CA, USA), using an Omega Wax Fused Silica Capillary column (Length, 30 m 0.3×2 mm D_f_, 0.25 μm, Sigma-Aldrich Co, St. Louis, MO, USA). Carrier gas and detector used was He gas and flame ionization detector, respectively.

### Statistical analysis

Data were analyzed using a MIXED procedure of SAS package program (SAS Inst. Inc., Cary, NC, USA) as a randomized completely block design. The model was,

$${\text{Y}}_{\text{i}(\text{t})}=\mu +{\text{B}}_{\text{i}}+{\text{T}}_{\text{ij}}+{\text{E}}_{\text{ij}(\text{t})},$$

where μ is average value, B_i_ is block, T_j_ is treatment value and E_ij(t)_ is the error value. The fixed effect feed treatment, and block was parity in procedure. Polynomial orthogonal contrasts were used to determine the fermentation effect according to time using the CONTRAST option. The crossing point of quadratic broken-line and the quadratic line was determined using NLIN code in order to determine proper fermentation time. Least squares mean between treatments were assessed using a pairwise comparison method. Statistical difference and tendency were accepted at p-value less than 0.05 and 0.10, respectively.

## RESULTS

### Chemical composition

Chemical composition of SICG relative to fermentation time is shown in Table 4. The DM content linearly decreased over fermentation time (p<0.001), as did the OM content (p = 0.008). The CP content showed no significant difference with fermentation time. The NDF and ADF content quadratically (p = 0.016) and linearly (p = 0.045) increased with fermentation time, respectively. The ADIN content linearly decreased (p = 0.037) with fermentation time.

### Microbial count and fermentation quality

Microbial count and fermentation characteristics in SICG in response to fermentation time are shown in Table 5. The microorganism counts increased linearly for *L. plantarum*, *S. cerevisiae*, and *B. subtilis* with fermentation time (p<0.001). In fermentation quality, pH content quadratically decreased (p = 0.026), lactic acid quadratically increased (p = 0.022), acetic acid linearly increased (p = 0.002), and butyric acid linearly increased (p<0.001) relative to fermentation time. However, propionic acid and ammonia nitrogen exhibited no difference during fermentation.

### Dry matter intake, milk yield, and milk composition

Dry matter intake (DMI), milk production and milk composition are shown in Table 6. The DMI of control and FSICG-fed cows showed no significant difference between treatments. Milk yield, 4% fat corrected milk (FCM), fat-protein corrected milk (FPCM) and feed to milk conversion content were also not significantly different between treatments. In the milk composition, fat, protein, lactose, non-fat solids, milk urea nitrogen, and somatic cell counts showed no significant difference between treatments.

### Blood profile

Blood profile is shown in Table 7. White blood cell (WBC) content was not significantly different between treatments. In terms of WBC composition, lymphocyte and granulocyte content were not significantly different between treatments, while the monocyte content of the control group was significantly greater than those of FSICG (p = 0.039). Red blood cell, hemoglobin, and platelet content showed no significant difference between treatments.

## DISCUSSION

In the fermentation trial, DM content decreased both linearly and quadratically relative to the fermentation time. Meanwhile, the OM content decreased only linearly relative to the fermentation time. These results are likely due to decreased nutrient composition via microbial fermentation. In the SICG, it was reported to have low available nutrients [4] which including the complex melanoidin which is difficult to break down [15]. For this reason, if the fermentation time is longer, a quadratic decrease in DM and OM content might be expected by the fermentation. The NDF and ADF content increased due to fermentation which can be explained as the increase in the proportion of NDF and ADF that would occur as the DM content decreases. As a result, it is evident that the fiber source of SICG was difficult to utilize by the microorganisms. The CP content was not significantly different after fermentation. However, ADIN content linearly decreased with fermentation time. This suggests that nitrogen could be utilized in the melanoidin complex. Furthermore, another nitrogen source was not added during the fermentation process in this study. In a previous study, it was reported that the fermentation using lactic acid bacteria could improve nitrogen utilization of SICG by sheep [8].

The number of *L. plantarum*, *S. cerevisiae*, and *B. subtilis* linearly increased over the entire fermentation time (p<0.001). Although the amount measured at inoculation of 0 d was lower than the target amount, the fermentation process proceeded without problems due to growth of the microbes. The pH result was quadratically decreased by the fermentation. Generally, fermentation has been used in the feed industry to prevent spoilage by microbes. The fast drop in pH is useful to prevent spoilage by unidentified microorganisms and can help to reduce the loss of DM by fermentation [16]. In this study, as the level of pH in FSICG was 4.43 to 4.79 after 3 d fermentation, it seems that it achieved a proper pH level to prevent spoilage. In the previous study, it reported that acidification caused by lactic acid was insufficient to prevent the growth of spoilage microbes, and the presence of other organic acid was needed [17]. As the aerobic stability is dependent on acetic acid concentration [18], the hetero-inoculation of this study seems that have a positive effect on the concentration of acetic acid. As both the lactic- and acetic acid content significantly increased in this study, it seems this could be a sufficient condition to prevent spoilage. Furthermore, the low level of butyric acid content in this study means there was not only good growth of target microorganisms but also the prevention of spoilage by microbes such as clostridium [19]. The high level of ammonia nitrogen in the fermented product has also been reported in association with clostridium fermentation [20]; however, in this study the ammonia nitrogen content was low compared with the previous study, and so not a level for concern.

The FSICG used in the animal trial was fermented for 14 days and was determined using DM, ADIN, microbial count, and lactic acid content. In the fermentation result, the crossing point between DM content (quadratic broken-line, p<0.001; quadratic line, p<0.001) and lactic acid content (quadratic broken-line, p = 0.004; quadratic, p<0.001) was shown at 9 days (not presented in table). The crossing point of quadratic broken-line in the lactic acid bacterial count (quadratic broken-line, p = 0.028; quadratic, p<0.001) was shown at 12 days (not presented in table). For these reasons, the minimum fermentation time was determined as 12 days. Furthermore, as ADIN content was shown to linearly decrease during fermentation time up to 21 days, protein availability was expected to be enhanced according to increased fermentation. However, increasing the fermentation time has the disadvantage of decreasing available nutrients [16]. In conclusion, the determined fermentation time was considered appropriate.

In the animal trial, FSICG diet did not effect the DMI. In the *ad*-*libitum* condition, although animals were included based on the consideration of milk yield, days in milk, and parties, it was considered that the daily feed intake might be not similar among animals. However, observation during the experimental period did not show any rejection of the treatment feed intake by the experimental animals. This in spite of a previous study which found adding wet coffee ground to the experimental feed resulted in the DMI being quadratically decreased in sheep [21], there was also no significant difference between SCG 0% and 10% treatments [8,21]. In this study, as the only 5% of cottonseed was replaced by FSICG in the feed, it was considered that have a small effect on the DMI.

All variables investigating milk production did not sig nificantly differ between the control and FSICG treatments. Although it has no significant difference in milk yield, 4% FCM, and FPCM, the value of the control was greater than those of FSICG treatment. This seems to have been influenced by the difference in DMI. However, the result was the opposite to those of feed to milk conversion. In the previous study in sheep, it was reported that as the FSICG level increased in the experimental feed, the gain to feed ratio decreased [8]. The difference between the previous and the current experiment seems to be due to the difference of replacement feed components. In the previous study, the alfalfa and timothy forage were replaced with FSICG in the experimental feed based on gross energy, whereas it was cottonseed that was replaced by FSICG in the current study. According to the calculation of digestible energy using [22], the replaced forage source was greater than those of the FSICG in the previous study, while the replaced cottonseed in the current study was similar to that of the FSICG. There were also no significant differences between control and FSICG treatments in milk chemical composition, similar to those of milk production. It means the replacement of 5% FSICG level in dairy feed might have little possibility of causing a negative effect on milk composition. In conclusion, cottonseed could be sufficiently be replaced by FSICG in ruminant feed.

Most of the blood profiles showed no difference between control and FSICG treatments except for monocyte content. The monocyte concentration in blood has been reported to range from 2.0% to 6.7% of WBC (Leukocytes, 4.9 to 13.3× 10^3^/μL; monocyte 0.1 to 0.8×10^3^/μL) [23], and so were in the normal range in this study. Although there was a significant difference between monocyte content of controls and the FSICG treatment, it was considered to not indicate a negative effect on the dairy cows as there was no significant difference in WBC content and they were within a reasonable range [23, 24]. As all of blood profiles were not significantly different between control and FSICG treatments, the FSICG does not seem to have a negative effect on blood profiles in dairy cows.

As the feeding of SICG in sheep has had a significant effect on water intake and urine excretion due to caffeine [8], the feeding of FSICG could be worrisome in dairy cows. In a previous study, fermentation using anaerobic microorganism has reported reduced caffeine concentration [25], with the highest reduction in the treatment with 5% molasses [26]. Furthermore, fermentation using microorganisms have found that caffeine can be reduced by 40% after 9 days fermentation [25]. Fortunately, the caffeine concentration in the 5% of FSICG feed is considered acceptable compared with previous studies because cattle are known to have high caffeine tolerance. Therefore, the fermentation could have a positive effect on the improvement of protein utilization and FSICG can be considered a substitute for cottonseed in the dairy cows as a feed component.

## IMPLICATIONS

The fermentation characteristics of SICG and the effect of FSICG on milk production, composition, and blood profiles were evaluated in dairy cows. Fermentation of SICG decreased DM, OM, ADIN, and pH content and increased microbial counts, lactic acid, and acetic acid content. While decreasing OM content reduced the feed energy value, decreasing ADIN may enhance nitrogen availability. The animal trials showed that 5% DM of FSICG had no negative effect on milk production and composition. Therefore, FSICG is a satisfactory substitute for cottonseed as a feed component, and 5% DM of dietary FSICG was appropriate for dairy cow diets.

## Figures and Tables

**Table 1:** Chemical composition of spent instant coffee grounds

Items	SICG
DM (%)	46.9±0.021)
OM (% DM)	98.2±0.03
CP (% DM)	10.9±1.30
EE (% DM)	11.2±0.05
NDF (% DM)	70.6±2.53
ADF (% DM)	66.3±1.12
ADIN (% DM of total N)	68.8±2.72

SICG, spent instant coffee grounds; DM, dry matter; OM, organic matter; CP, crude protein; EE, ether extract; NDF, neutral detergent fiber; ADF, acid detergent fiber; ADIN, acid detergent insoluble nitrogen.

1) Mean±standard deviation.

**Table 2:** Chemical composition of fermented spent instant coffee grounds after 14 days fermentation

Items	SICG1)	FSICG1)
Chemical compositions		
OM (% DM)	98.30±0.43	96.91±0.082)
CP (% DM)	10.50±0.53	11.72±0.07
NDF (% DM)	70.33±0.33	74.82±0.72
ADF (% DM)	59.67±0.14	62.87±0.09
ADIN (% DM of total N)	70.67±0.01	66.14±0.01
Cells count (Log_10_cfu/g)		
*Lactobacillus plantarum*	ND	5.43±0.18
*Saccharomyces cerevisiae*	ND	5.68±0.38
*Bacillus subtilis*	ND	5.46±0.23
Fermentation characteristics		
pH	5.38±0.11	4.54±0.07
Lactic acid (μg/L)	ND	4.92±0.01
Acetic acid (μg/L)	ND	1.05±0.02
Propionic acid (μg/L)	ND	0.13±0.00
Butyric acid (μg/L)	ND	0.00±0.33
Ammonia-N (μg/mL)	ND	2.30±0.03

OM, organic matter; DM, dry matter;CP, crude protein; NDF, neutral detergent fiber; ADF, acid detergent fiber; ADIN, acid detergent insoluble nitrogen; ND, not detected.

1) SICG (spent instant coffee ground), not fermented spent instant coffee grounds+5% molasses; FSICG (fermented spent instant coffee ground), fermented spent instant coffee grounds+5% molasses.

2) Mean±standard deviations.

**Table 3:** Total mixed ration formula and chemical composition of diets in fermented spent instant coffee ground feeding trial in lactating dairy cows

Items	Control	Treatment
Ingredient (%)		
Commercial mixed feed1)	20.83	20.83
Molasses	2.22	2.22
Corn cracked	6.11	6.11
Corn gluten feed	4.17	4.17
Beet pulp pellet	1.39	1.39
Brewers grain (wet)	9.03	9.03
Cotton seed	6.25	4.86
Cotton seed pellet	1.39	-
FSICG	-	10.56
Alfalfa hay	8.33	8.33
Oat hay	4.17	4.17
Timothy hay	5.56	5.56
Bermuda grass hay	2.78	2.78
Klein grass hay	4.86	4.86
Water	22.92	15.14
Total	100.00	100.00
Chemical composition		
DM (%)	62.41	63.63
Forage ratio (% DM)	37.03	36.32
TDN (% DM)2)	67.88	68.54
CP (% DM)	16.57	16.34
EE (% DM)	4.40	4.59
NFC (% DM)	29.27	28.48
CF (% DM)	18.49	18.67
NDF (% DM)	42.49	43.65
ADF (% DM)	25.27	26.62
NE_L_ (Mcal/kg DM)2)	1.54	1.56

FSICG, fermented spent instant coffee grounds; DM, dry matter; TDN, total digestible nutrient; CP, crude protein; EE, ether extract; NFC, non-fibrous carbohydrate; NDF, neutral detergent fiber; ADF, acid detergent fiber; NE_L_, net energy for lactation.

1) Commercial mixed feed formula: Corn grain, 30.0%; molasses, 5.0%; soybean meal, 22.2%; rapeseed meal, 7%; corn gluten feed, 10.0%; copra meal, 5.6%; palm oil meal, 15.0%; limestone, 2.3%; salt, 0.8%; sodium bicarbonate, 0.8%; by-pass fat, 0.3%; Vit and mineral premix, 1.0%.

2) TDN and NE_L_ was calculated by NRC 2001 model.

**Table 4:** Changes of chemical composition in spent instant coffee grounds according to fermentation time

Chemical compositions	Time (d)							SEM	p-value1)	
	
	0	3	6	9	12	15	21		L	Q
DM (%)	34.1	31.5	30.0	29.9	29.7	29.2	28.2	0.019	<0.001	<0.001
OM (% DM)	97.4	96.9	96.9	96.9	97.0	96.7	96.8	0.107	0.008	0.175
CP (% DM)	10.5	11.7	12.1	11.4	12.1	12.0	12.5	0.723	0.131	0.635
NDF (% DM)	70.3	73.1	75.3	74.3	73.6	73.3	73.0	0.857	0.240	0.016
ADF (% DM)	59.7	62.1	62.8	62.9	62.6	62.9	63.0	0.833	0.045	0.097
ADIN (% DM of total N)	84.7	83.3	81.5	80.8	79.6	75.3	68.0	5.224	0.037	0.500

SEM, standard errors of the mean; DM, dry matter; OM, organic matter; CP, crude protein; NDF, neutral detergent fiber; ADF, acid detergent fiber; ADIN, acid detergent insoluble nitrogen.

1) L, linear effect; Q, quadratic effect.

**Table 5:** Changes of microorganism counts and fermentation characteristics in spent instant coffee grounds according to fermentation periods

Items	Time (d)							SEM	p-value1)	
	
	0	3	6	9	12	15	21		L	Q
Microbial counts (Log_10_cfu/g)										
*Lactobacillus plantarum*	4.58	4.86	5.68	5.18	6.07	5.87	6.79	0.176	<0.001	0.993
*Saccharomyces cerevisiae*	4.67	5.17	5.87	5.49	6.20	5.81	6.76	0.202	<0.001	0.508
*Bacillus subtilis*	4.96	4.96	5.66	5.26	5.98	5.62	6.72	0.188	<0.001	0.244
Fermentation characteristics										
pH	5.38	4.43	4.51	4.58	4.52	4.77	4.79	0.071	0.050	0.026
Lactic acid (μg/L)	1.68	2.83	3.88	4.24	5.65	5.17	6.13	0.370	<0.001	0.022
Acetic acid (μg/L)	0.77	0.78	0.81	0.86	1.00	1.03	1.29	0.118	0.002	0.323
Propionic acid (μg/L)	0.01	0.01	0.01	0.26	0.00	0.00	0.02	0.070	0.837	0.130
Butyric acid (μg/L)	0.01	0.00	0.00	0.00	0.00	0.00	0.03	0.001	<0.001	<0.001
Ammonia-N (μg/mL)	1.33	2.67	3.06	1.56	1.56	1.72	2.83	0.987	0.761	0.751

SEM, standard errors of the mean; cfu, colony forming unit

1) L, linear effect; Q, quadratic effect.

**Table 6:** Dry matter intake, milk production and composition of fermented spent instant coffee ground feeding trial in lactating dairy cows

Items	Control	FSICG	SEM	p-value
Dry matter intake (kg/cow/d)	27.74	26.11	1.19	0.210
Milk production				
Milk yield (kg/cow/d)	31.00	30.22	2.45	0.368
4% FCM1) (kg/cow/d)	33.82	32.14	2.84	0.262
FPCM2) (kg/cow/d)	32.06	31.26	0.80	0.364
Feed to milk conversion (kg/kg DM)	1.12	1.16	0.09	0.297
Milk chemical composition				
Fat (%)	4.59	4.43	0.20	0.199
Protein (%)	3.32	3.20	0.13	0.157
Lactose (%)	4.65	4.80	0.10	0.077
Solid not fat (%)	8.65	8.65	0.15	0.497
Milk urea nitrogen (mg/dL)	14.01	14.80	1.08	0.275
Somatic cell counts (10^3^ cells/mL)	103.44	59.75	33.28	0.081

FSICG, fermented spent coffee ground; SEM, standard error of the means; FCM, fat corrected milk; FPCM, fat-protein corrected milk; DM, dry matter.

1) 4% FCM was calculated from 4% FCM = 0.4×milk yield+15×milk fat yield.

2) FPCM was calculated from FPCM = milk yield×(0.337+0.116×milk fat [%]+0.06×milk protein [%]).

**Table 7:** Blood profiles of fermented spent instant coffee ground feeding trial in lactating dairy cows

Items	Control	FSICG	SEM	p-value
White blood cell (10^3^/μL)	11.52	11.51	1.046	0.996
Lymphocyte (% of WBC)	55.82	61.62	3.898	0.309
Monocyte (% of WBC)	6.12	4.03	0.66	0.039
Granulocyte (% of WBC)	38.06	34.37	3.997	0.523
Red blood cell (10^6^/μL)	6.40	6.72	0.295	0.452
Hemoglobin (g/dL)	11.73	12.28	0.313	0.232
Platelet (10^3^/μL)	349.22	415.67	47.846	0.341

FSICG, fermented spent coffee ground; SEM, standard error of the means; WBC, white blood cell.
